# “Constipation” of Nervous Origin

**Published:** 1883-08

**Authors:** 


### "CONSTIPATION" OF NERVOUS ORIGIN.

THERE are many diverse forms or causes of this malady—or abnormity of function—which it is important to recognize, but which are too commonly confounded, with the inevitable result of failure in the precise direction of remedies. The following may be noted as two or three of the commonest varieties of the trouble. For example, defect of muscular movements may be the cause of inaction of the bowels. This may be either want of peristaltic action in the small intestines or of extrusive force in the muscles concerned in the act of defecation; the location of the inertia being determinable by the character of the fæces, and the period and manner of their dejection. The characteristics of fæces recently discharged from the small intestines and rapidly passed through the large intestine are two familiar to call for description. The scybalous dejecta of long retained fæces are equally well known. The error, if error there be, which is made in practice relates to the neglect of such lessons as the characteristics of the fæces are intended to teach, in regard to the cause or form of the "constipation." We lay great stress on this matter in diarrhœa, but it is practically disregarded in consumption, whereas, if possible, the character of the dejecta is more suggestive as an indication of the place and cause of detention than as an indication of the nature and cause of excessive or too rapid discharge. It is, for obvious physiological reasons, of the highest moment to determine whether constipation be due to inactivity of the muscular coats of the intestine or of the levator ani and accessory muscles concerned in the act of evacuation. Second, there may be, and often is, instead of inactivity, irritability of certain muscles as a cause of constipation. In a considerable proportion of cases of constipation with loaded rectum, the actual cause of the retention of the fæces is spasmodic contraction of the sphincter, which occurs the moment the levator ani begins to act. Only a little of the watery portion of the contents of the bowel escapes, the scybalous mass being retained. In cases of this class there is apt to be considerable disturbance of the pelvic viscera and other discharges while at stool. The cause of these muscular troubles is of course located in the nervous system. Sometimes the reflex excitability which should respond to the presence of fæces at particular parts of the intestinal canal is abnormally depressed, as in the constipation which arises from certain tabetic forms of spinal disease. In others the reflex irritability is unduly exalted, as in the cases of spasmodic contraction of the sphincter of which we have spoken. This increased excitability in the lower part of the cord is common, and it is generally to spasmodic irritability of the sphincter those forms of constipation are due in which the use of tobacco affords temporary relief, and seems to produce an action. As a rule it may be assumed that "habitual constipation" is an affection the cause of which must be sought in spinal exhaustion or irritability, or the two states combined. In another group of cases the cause of constipation is inactivity of the secreting glands of the intestines, from defect of innervation. We are not speaking of the cases in which the larger viscera, such as the liver, may be at fault; but of a multitude of instances in which the glandular system and apparatus of the walls of the intestinal canal are not in such a state of erectile and functional activity as to maintain the reflex excitability of the parts, and to so lubricate the contents of the canal as to pass them rapidly along in course. This is *one* of the causes of that broken earth-like type of dejecta which attends a form of constipation common among the subjects of spare and shriveled physique, who are insensitive to most external impressions though, perhaps, preternaturally sensitive to some special excitants. It is not within the scope of our purpose to discuss this topic in detail, but it is one to which the attention of the profession might be usefully directed. The practice of treating "habitual constipation" by cathartics is almost played out, and it is time the study of this common and most intractable affection from the standpoint of nervous origin and causation should be seriously and earnestly undertaken.

